# 

**Published:** 1922-07

**Authors:** 


### THE MONTH IN PARLIAMENT.

May 29.—The financial resolution on the National Health Insurance Bill was taken. In the course of the debate Sir G. Collins inquired whether any progress had been made with the consolidation of the Insurance Acts foreshadowed last year by the Minister of Health. Sir A. Mond replied that the matter was being carefully studied, but that he could not say when legislation would be introduced for the purpose.

June 13.—The Ministry of Health vote was taken. Sir A. Mond, speaking on his Department's estimates, pointed out that he had been able to do even better than the recommendations of the Geddes Committee, the total vote being £22,062,000, or £69,000 less than that Committee had suggested as a reasonable sum. He explained the reductions which had been made and dealt at length with the housing situation. The price of houses was now coming down to an economic level, and private enterprise would soon enter the field again. He then referred to the hospital question, and spoke warmly of the work done by Lord Cave's Committee and subsequently by the Voluntary Hospitals Commission presided over by Lord Onslow, which administers the Exchequer grant of £500,000 voted to meet the more urgent needs of the hospitals. He mentioned that 42 hospital committees are now working in England, Scotland and Wales, and six will shortly be working. The mass-contribution schemes were showing remarkable results. Mr. Trevelyan Thomson moved to reduce the vote by £100, and criticised the Government's housing policy. Mr. Cautley and others continued the debate, and ultimately progress was reported, the further discussion of the vote being adjourned.

The Audit (Local Authorities, etc.) Bill has been passed by the House of Lords without amendment, and awaits the Royal Assent.

The National Health Insurance Bill received a second reading in the House of Commons, and was referred to a Standing Committee.

The Milk and Dairies (Amendment) Bill was read a first time in the House of Lords on June 20.

### Approved Societies and Hospital Treatment.

*To the Editor of* The Hospital and Health Review.

### RECENT APPOINTMENTS.

### Public Health.

Dr. George Cockrame, of Buxton, to be M.O.H. for the Rural District of Chapel-en-le-Frith.

Mr. F. G. E. Hill, M.B., Ch.B., D.P.H., D.S.O., to be Assistant School Medical Officer to the City of Hull Education Committee.

Dr. James Edwin Wilson, M.B., M.D., B.Ch., B.A.O., D.P.H., late Deputy M.O.H., Grimsby, to be M.O.H. and S.M.O. for the Borough of Mansfield.

Dr. W. T. Kilgour, M.R.C.S., L.R.C.P., formerly Assistant Tuberculosis Officer for Stepney Borough, to be Assistant Tuberculosis Officer for the County Borough of West Ham.

Mr. Bertram Conrad Haller, M.A., L.R.C.P., L.R.C.S., D.P.H., at present Assistant to M.O. for Maternity and Child Welfare, Aberdeen, and formerly of the R.A.M.C., to be Assistant Medical Officer of Health for the Borough of Wolverhampton.

### Institutional.

The new secretary of Harrow Cottage Hospital is Captain Lionel Hewlett, who succeeds the late Mr. B. P. Lascelles.

Mr. E. F. Hunt, honorary secretary of the Acton Hospital for twenty-one years, has been re-elected chairman.

Mr. J. J. Turner, assistant secretary to the Carlisle Fever Hospital, has been appointed to succeed the late Mr. James Lovett, who held the post of honorary secretary for thirty-six years.

Dr. Owen F. McCarthy has been appointed Medical Superintendent of Cork Mental Hospital, in the service of which he has been for twenty-one years.

Dr. O. G. Connell, M.C., has been appointed medical superintendent of the Norfolk County Mental Hospital. Born in 1885, Dr. Connell qualified in 1910, and was senior medical officer of Salop Mental Hospital, and assistant at Middlesex County Mental Hospital, Napsbury. In 1919 he was in charge of a section of the military Neurological Hospital, Ashurst, Oxford. He served on almost every front during the war.

Dr. E. L. White, formerly Assistant Medical Officer of Health for the County Borough of Southampton, to be Medical Superintendent of Shirley Warren Infirmary, Southampton.

Dr. Richard C. Hutchinson, late Medical Superintendent of Baguley Sanatorium, Timperley, to be Medical Superintendent of the Royal National Hospital for Consumption, Ventnor.

### VENEREAL DISEASE STATISTICS.

It must be evident even to the layman, says a report published by the United States Public Health Service, that the damage done by venereal disease is enormous. Unfortunately, concealment is so general that there is little dependable information yet available. For improvement in reporting cases and in giving the true causes of deaths reliance must be had on the efforts of workers in the syphilitic field. They must become missionaries in popularising the notification required by law. Half the doctors in the United States have agreed to co-operate with the State Boards of Health in this respect, and the great majority of the other half would fall into line if they were convinced that co-operation would protect their patients and that it was really wanted by the community. This would give much-desired information as to the real prevalence of venereal diseases.

Meanwhile the Public Health Service suggests that doctors whose practice has brought them into contact with large numbers of syphilitic patients over long periods of time might send to some central office the histories of their patients from the time of infection, through the period of treatment, to dismissal either through death or clinical recovery. Army and Navy medical records should also be valuable, for both contain large numbers of records of the infection of personnel. Combined study of these should disclose the actual mortality from syphilis and the impairments, or sequelæ, which have resulted from it. A large series of records might conceivably show a fairly constant relationship between syphilitic infection and aneurisms, locomotor ataxia, general paralysis, organic heart disease, and other conditions. For such a study a large number of cases, widely distributed and checked by statistical requirements of age, sex, and race, would be necessary.

### OFFICIAL REPORTS RECEIVED.

The College of Nursing, Ltd.
Report of the Executive Committee of the Nation's Fund for Nurses (Regd.).
Weekly Reports of the United States Public Health Service, Vol. 37, Nos. 15-20.
Urban District of Chadderton: Dr. James Wood (M.O.H. and S.M.O.).
Metropolitan Borough of Poplar: Special Report, "A Suggestion—How to Prevent Cancer." By Dr. F. W. Alexander (M.O.H.).
Corporation of Glasgow: Report by Medical Officer of Health (Dr. A. K. Chalmers) on the Food Value of Diets supplied under the Maternity and Child Welfare Scheme.
Dr. E. V. Shaw's Report to the Ministry of Health on an Epidemic of Enteric Fever at Bolton-upon-Dearne. (Reports on Public Health and Medical Subjects, No. 12. H.M. Stationery Office, 2s. net.)

**Hospitals and Homes (year to Dec. 31, 1921, unless otherwise stated):—**

Royal Waterloo Hospital for Children and Women.
St. Mary's Hospital, Paddington.
Batley and District Hospital.
North Ormesby Hospital, Middlesbrough.
Rochdale Infirmary and Dispensary.
West London Hospital, Hammersmith.
Devonshire Hospital and Buxton Bath Charity, Buxton.
Radcliffe Infirmary and County Hospital, Oxford.
Seamen's Hospital Society ("Dreadnought"), Greenwich.
Manchester Royal Infirmary, Dispensary and Royal Lunatic Hospital or Asylum.
County and City of Worcester Mental Hospital, Powick. Year to March 31, 1922.
West Kent General Hospital, Maidstone.
Royal Hospital for Sick Children, Glasgow.
Royal Alexandra Infirmary, Paisley (late Paisley Infirmary).
Glasgow Royal Infirmary.
Royal National Orthopædic Hospital.
Cumberland and Westmorland Mental Hospital.
Mental Hospital for the City of Leicester, West Humberstone. Year to March 31, 1922.

### GOVERNMENT PUBLICATIONS.

[Copies can be purchased through any bookseller, or direct from H.M. Stationery Office, at Imperial House, Kingsway, W.C.2; 28, Abingdon Street, S.W.1; 37, Peter Street, Manchester; 23, Forth Street, Edinburgh; and 1, St. Andrew's Crescent, Cardiff. The prices quoted include postage.]

### New Bills, &c.

Merchant Shipping (Venereal Disease) (No. 2).—To amend the Merchant Shipping Acts, 1894 to 1921, with respect to seamen suffering from venereal disease. 2½d.
Bread Acts Amendment.—(As amended by Standing Committee A). 2½d.
Sale of Tea.—To provide for the better protection of the public in relation to the sale of tea. 2½d.

### National Health Insurance.

Deposit Contributors Amendment Regulations, May 8, 1922, amending the Regulations 1918 to 1920. 1½d.
Report by Government Actuary on Valuation of Assets and Liabilities of Approved Societies as at Dec. 31, 1918. 4s. 2½d.

### Miscellaneous Reports and Papers.

Report of Fuel Research Board on Low Temperature Carbonisation. 2s. 3d.

---

The proprietors of "Glaxo" have just issued a revised (14th) edition of their "Baby Book," which is attractively printed and illustrated, and contains advice, simply worded, on points likely to arise in the rearing of infants and young children. It can be obtained, price 1s. 2d., post free, from Glaxo House, 56 Osnaburgh Street, London, N.W. 1.

### DIARY OF THE MONTH.

The International Union Against Tuberculosis, 20 Hanover Square, W. 1, will hold its third international conference at Brussels on July 11 to 13. The subjects for discussion are: (1) Tuberculosis in the child before and during school life; (2) anti-tuberculosis prophylaxis in the home by the visiting nurse, (3) the work of the tuberculous during and after cure. Visits will be organised to anti-tuberculosis establishments in Belgium.

The thirty-third Congress and Health Exhibition of the Royal Sanitary Institute will be held at Bournemouth from July 24 to 29, under the presidency of Major-General J. E. B. Seely, M.P. Delegates have been appointed by the Board of Control, the Ministry of Pensions, the Office of Works, the War Office and the Scottish Board of Health, and 23 Dominion and foreign Governments and municipalities are sending representatives.

The Voluntary Hospitals Commission will hold a conference of representatives of Local Hospital Committees on July 18 and 19, at the Ministry of Health. It is hoped that all local hospital committees will be represented by their chairman and secretary, but other members of committees will be welcome. Cards of admission will be sent only to those persons whose names have been notified to the Commission not later than July 10.

A conference will be held at Caxton Hall, Westminster, on July 26 and 27, under the auspices of the Central Association for Mental Welfare (formerly the Central Association for the Care of the Mentally Defective). The first day will be devoted to discussion of "Mental Deficiency in Relation to Crime," and Lord Justice Atkin will give a concluding address. The second day, when Mr. H. A. L. Fisher will address the conference, will be devoted to educational matters. Tickets (2s. 6d.) may be obtained from the Hon. Secretary, C.A.M.W., 24 Buckingham Palace Road, S.W.1. A full report of the Conference, including papers and discussions, will be sold at a cost of 3s., post free. Orders should be placed as soon as possible.

The Sanitary Inspectors' Association has issued the preliminary programme of its 35th annual conference, to be held at Buxton on September 5-8. The subjects to be discussed include the administration of the law relating to the sale of milk, the reports of the Departmental Committees on Meat Inspection and on Smoke Abatement, and the problems arising out of the present shortage of houses. An excursion will be made to Haddon Hall and Chatsworth House. The fee for members and associates of the S.I.A. is 15s. Notices of attendance should be sent to the hon. secretary at 15, Bessborough Street, London, S.W., as soon as possible.

A conference on birth control, convened by the Mayor of Deptford, is to be held at the Town Hall, New Cross Road, S.E. 14, on July 10, at 8.30 p.m. A paper will be read by Dr. C. S. Thomson, Medical Officer of Health for Deptford.

---

We are requested by the Dental Board of the United Kingdom to remind those who desire to obtain registration or entry under one of the various sections in the Dentists Act, 1921, that application should be made as quickly as possible. Under Section 1 (4) of the Act application must be made within the interim period defined in Section 14. About two months is occupied between the receipt of an application and the completion of registration, and a person who is not registered when the Act comes into force, whether he has made application or not, will be prohibited from practising.

---

The Industrial Hygiene Section of the International Labour Office, Geneva, proposes to publish from time to time a series of notes regarding new publications dealing with industrial hygiene. It would be grateful if members of the medical profession and others interested in problems of industrial health would send particulars of such publications, including articles in periodicals, to "The Industrial Hygiene Section, International Labour Office, Geneva, Switzerland," giving author's name, title of book or article, and publisher's name or, in the case of an article, the name, date and number of the periodical in which it appears. Correspondents will be supplied with the bibliographical notes as they are issued.

